# Cytotoxicity of Selected Nanoparticles on Human Dental Pulp Stem Cells 

**DOI:** 10.22037/iej.2017.28

**Published:** 2017

**Authors:** Kasra Tabari, Sepanta Hosseinpour, Peter Parashos, Parisa Kardouni Khozestani, Hossein Mohammad Rahimi

**Affiliations:** a * Dental Research Center, Research Institute of Dental Sciences, Dental School, Shahid Beheshti University of Medical Sciences, Tehran, Iran; *; b * Research Fellow, Dental Research Center, Research Institute of Dental Sciences, Students' Research Committee, Shahid Beheshti University of Medical Sciences, Tehran, Iran; *; c * Melbourne Dental School, University of Melbourne, Melbourne, Victoria, Australia; *; d *Pathology Department, Dental School, Gilan University of Medical Sciences, Rasht, Iran; *; e * Students' Research Committee, Dental School, Shahid Beheshti University of Medical Sciences, Tehran, Iran*

**Keywords:** Cytotoxicity, Dental Pulp Stem Cells, Metal Oxide Nanoparticle

## Abstract

**Introduction:**

Nanoparticles are being increasingly applied in dentistry due to their antimicrobial and mechanical properties. This *in vitro* study aimed to assess and compare the cytotoxicity of four metal oxide nanoparticles (TiO_2_, SiO_2_, ZnO, and Al_2_O_3_) on human dental pulp stem cells.

**Methods and Materials:**

Four suspension with different concentrations (25, 50, 75, 100 µg/mL) of each nanoparticle were prepared and placed into cavities of three 96-well plates (containing 1×10^4^ cells per well that were seeded 24 earlier). All specimens were incubated in a humidified incubator with 5% CO_2_ at 37^°^C. Mosmann’s Tetrazolium Toxicity (MTT) assay was used to determine *in vitro* cytotoxicity of test materials on pulpal stem cells. Cell viability was determined at 24, 48, and 72 h after exposure. Data comparisons were performed using a general linear model for repeated measures and Tukey's post hoc test. The level of significance was set at 0.05.

**Results:**

The tested nanoparticles showed variable levels of cytotoxicity and were dose and time dependant. The minimum cell viability was observed in ZnO followed by TiO_2_, SiO_2_ and Al_2_O_3_.

**Conclusion:**

The results demonstrated that cell viability and morphological modifications occurred at the concentration range of 25 to 100 µg/mL and in all nanoparticles. The higher concentration and longer duration of exposure increased cellular death. Our results highlight the need for a more discrete use of nanoparticles for biomedical applications.

## Introduction

Biomedical administrations of nanoparticles vary from gene/drug delivery and malignancy therapies to dental applications [1-3]. Nanotechnology presents a wide diversity of nanomaterial especially inorganic nanoparticles based on metal oxides and quantum dots with different morphologies like tubes, spheres, prisms and rods [4-7]. 

In dentistry, nanoparticles are increasingly applied due to their antimicrobial and mechanical properties [8-10]. TiO_2_ nanoparticles are used in manufacturing of dental materials for their antibacterial activity, being chemically inert, low price, high resistance and hardness [11-13]. Previous studies demonstrated anti-biofilm activity of ZnO nanoparticles against *Staphylococcus aureus* and *Enterococcus* *faecalis* after their inclusion in mineral trioxide aggregate (MTA) formulations [14-16]. However, it is reported that the presence of ZnO nanoparticles can reduce the compressive strength of Portland cement [14]. On the other hand, addition of Al_2_O_3 _nanoparticles can increase flexural and tensile strength of composites [17]. Combination of nano CaO, Al_2_O_3 _and white MTA improves biological and sealing properties of MTA and also reduce its setting time [18, 19]. Furthermore, SiO_2_ at low content (2 wt%) significantly intensified the mechanical properties of polyhedral oligomeric silsesquioxane nanocomposite and with the enhancement of its content, the mechanical properties declined [20]. 

The current expeditious growing interest in nanoparticles for biomedical applications progressively necessitates their toxicity evaluations. Despite of the above advantages, there are concerns regarding their potential adverse impact on organisms. Previous studies also indicated some toxic effects of nanoparticles [21-23] such as apoptotic and micronuclei inductive impact of nano TiO_2_ [24], DNA damage and IL6 secretion enhancement by nano SiO_2_ [25], and decreased viability of human lung epithelial cells and proliferation with addition of ZnO and Al_2_O_3_ [26]. Nevertheless, there is limited comparative information about the nanoparticles and their effects on human dental pulp stem cells (DPSCs). DPSCs are the initiative cell sources for differentiation of odontoblasts like cells to produce reparative dentin especially in vital pulp therapy approaches [27]. 

The present article is the first part of our comprehensive study on evaluating and developing a novel nano incorporated MTA which is mainly intended to be used for direct pulp capping and vital pulp therapy. Thus, it is necessary to investigate the potential toxicity of nanoparticles on human DPSCs. 

The objectives of the present study were to specify whether nanoparticles of TiO_2_, SiO_2_, ZnO and Al_2_O_3_ affect the viability of DPSCs, to compare their cytotoxicity and to provide information for selecting the best cost effective nanoparticle to be utilized in formulation of nanohybrid MTA production as the next step.

## Materials and Methods

***Nanoparticles and specimen preparation***

Table 1 shows the properties TiO_2_, Al_2_O_3_, ZnO, and SiO_2_ nanoparticles that were used in this study. The powder forms of these materials were suspended in sterile distilled water at a concentration of 50 mg/mL. In order to prepare a homogenous suspension, they were sonicated (S‐4000 Sonicator ultrasonic processor, Misonix Inc., Farmingdale, NY, USA) at intensity of 33 W and 20 kHz frequency for 1 min. In order to prepare four concentrations of suspension of each material (25, 50, 75, 100 μg/mL), Dulbecco's modified Eagle's medium (DMEM) (D5030, Sigma-Aldrich, St. Louis, MO, USA) and 10% fetal bovine serum (FBS) (F6178, Sigma-Aldrich, St. Louis, MO, USA) were added and again sonicated for 2 min [28, 29]. 

***Cell culture***

For this *in vitro *cytotoxicity testing DPSCs were previously provided by flow cytometry and cell differentiation in Molecular and Cell Biology Laboratory of Dental Research Center, Shahid Beheshti University of Medical Sciences. After thawing process, DPSCs were cultivated in DMEM supplemented with 10% FBS, 100 U/mL penicillin, 100 μg/mL streptomycin, and 0.25 μg/mL amphotericin B (Sigma-Aldrich Corporation, St. Louis, MO), at 37^°^C in a 95% humidified atmosphere and 5% CO_2_ [30]. After reaching proper confluence and trypsinization, 1×10^4^ cell per well were gently seeded into 96-well plates (Costar, USA) and incubated for 24 h (at 37^°^C, 95% humidity and 5% CO_2_). Then, 200 μL of various concentration of suspensions and controls were replaced with the medium and the cells were incubated for 24, 48, and 72 h. Then 0.2% chlorhexidine was used as positive control and DMEM with the supplements as negative control. All experimental and control groups were performed in triplicate wells.

***Cell viability***

Dimethylthiazole-diphenyl tetrazolium bromide assay or Mosmann’s Tetrazolium Toxicity (MTT) assay was administrated for cytotoxicity evaluations. At each time point, the supernatant was rinsed by phosphate-buffered saline (PBS) (Gibco BRL, Grand Island, NY, USA). Then, 100 μL of MTT solution (Sigma-Aldrich, St. Louis, MO, USA) was added to each well. Plates were incubated for 3 h (at 37^°^C, 95% humidity and 5% CO_2_) to permit the viable cells to convert the soluble MTT salt (yellow) into insoluble crystals of formazan (purple). Next, the supernatant cells were discarded and 100 μL of dimethyl sulfoxide (DMSO, Gibco BRL, Grand Island, NY, USA) was added to each well for dissolving the formazan crystals. The remaining stain was measured by ELISA reader (Anthos 2020, NSW, Australia) at 570 nm wavelength with 650 nm filter to determine the percentage of cell viability. 

***Statistical analysis***

All data were analysed using SPSS 20.0.1 software (IBM, Armonk, NY, USA). After application of normality test on data, general linear model test for repeated measures ANOVA followed by the post-hoc Tukey’s tests were used for comparisons and the level of significance was set at 0.05.

## Results

***24 h after treatment***

**Figure 1:**
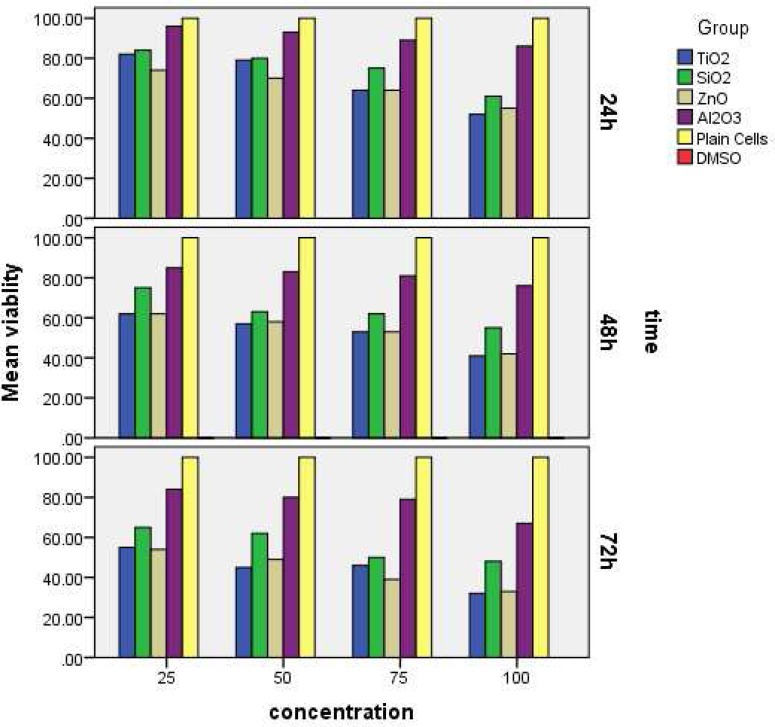
Percentage of DPSCs viability (mg/mL) after exposure to 25, 50, 75, and 100 μg/mL at: *A)* 24; *B)* 48 and *C)* 72 h

Figure 1A shows the difference in DPSCs viability between groups following exposure to 25, 50, 75, and 100 μg/mL suspensions of TiO_2_, Al_2_O_3_, ZnO, and SiO_2_ after 24 h. All differences between experimental and control groups were statistically significant (*P*<0.05) except between ZnO and TiO_2_ at 25 μg/mL concentration (*P*>0.05). While 90% of cell viability indicated non-toxicity, 60-90% indicated mild, 30-60% indicated moderate and less than 30%, indicated severe toxicity, respectively. Nanoparticles of Al_2_O_3_ were not toxic, and the others were mildly toxic compared with the negative control (*P*<0.05). Also, the toxicity followed a dose dependent pattern. 

***48 h*** ***after*** ***treatment***

Figure 1B shows the difference in DPSCs viability between groups following exposure to 25, 50, 75, and 100 μg/mL suspensions of TiO_2_, Al_2_O_3_, ZnO, and SiO_2_ after 48 h. All differences between experimental and control groups were statistically significant (*P*<0.05). Nanoparticles of Al_2_O_3_ were mildly toxic, and others were moderately toxic compared with negative control (*P*<0.05). Also, the toxicity showed a dose and time dependent manner. 

***72 h after treatment***

Figure 1C shows the difference in DPSCs viability between groups following exposure to 25, 50, 75, and 100 μg/mL suspensions of TiO_2_, Al_2_O_3_, ZnO, and SiO_2_ after 72 h. All differences between experimental and control groups were statistically significant (*P*<0.05). Although all cell viability decreased from 24 to 48 h, but none of the experimental groups showed severe toxicity. Moreover, the toxicity showed a dose and time dependent manner similar to the previous time points. 

## Discussion

Commercialization of nanoparticles for nanomedicine is rapidly increasing and many nanoparticle-containing products in the form of medicines, varnishes and cosmetic goods are available in the market [31]. Therefore, unexpected adverse effects of these particles is a growing concern in society [32] and academic environments [21, 33]. In the present study, we assessed and compared the *in vitro* cytotoxicity of TiO_2_, SiO_2_, ZnO, and Al_2_O_3_ nanoparticles on DPSCs. The results demonstrated that cell viability and morphological modifications occurred at the concentration range of 25 to 100 μg/mL and the toxicities are dose and time dependant in all four nanoparticles. The minimum cell viability was observed in ZnO followed by TiO_2_, SiO_2_, and Al_2_O_3_.

In accordance to our results, Dechsakulthorn *et al.* [34] indicated a dose dependent cytotoxicity of ZnO nanoparticles and TiO_2_ nanoparticles *via* Non-Radioactive Cell Proliferation (MTS) assay. Also, higher toxicity of ZnO nanoparticles compared to TiO_2 _nanoparticles was reported. Zheng *et al.* [35] reported that presence of nano ZnO significantly inhibited L929 mouse fibroblasts and Hela cells proliferation *via* MTT assay, cell flow cytometry, light and electron microscopy evaluations. Moreover, they feed 30 mice suspension of nano ZnO (30 mg/mL) through digestive tract and observed glomerular swelling in kidney, inflammation in heart and hydropic degeneration in liver. However, the combination of nano-scale ZnO in zinc-oxide eugenol sealer showed the same biocompatibility compared to Pulpdent (commercially available ZOE-based sealer) by MTT assay [36]. *In vivo *experiments demonstrated adverse effects of Zn and TiO_2_ nano-powder in mice at 5 g/kg body weight [37, 38] and pulmonary toxicity of TiO_2_ nanoparticles in rats at 5 mg/kg concentration after inhalation [39]. Inhalation is the most common route of exposure among these studies [40, 41] which is not necessarily important for dental material applications. For instance, Heravi *et al. *[42] reported lower toxicity of nano-scale TiO_2 _containing orthodontic adhesives compared with conventional adhesives and indicated that the addition of 1 wt% TiO_2_ nanoparticles into a conventional adhesive did not entail extra health concerns in comparison with conventional pure resin. 

Pakrashi *et al. *[43] demonstrated the cytotoxicity of Al_2_O_3_ nanoparticles for bacteria at very low concentration (less than 1 μg/mL). This fact together with our results can suggest the potential administration of this nanoparticle as an antibacterial agent in dentistry. In addition, even the biocompatibility of dental materials containing Al_2_O_3_ nanoparticles can be promoted by addition of hydroxyapatite [44]. 

In agreement with our results, Gong *et al.* [4] indicated the dose dependent reduction of cell viability after exposure to nano and micro sized SiO_2 _within HaCaT cells. However, Afsharnezhad *et al.* [45] showed that the application of SiO_2 _nanoparticles can even improve the level of biocompatibility of a cyanoacrylate composite in addition to promotion of its mechanical properties. 

**Table 1:** Nanoparticle characteristics

**Product name**	**Form **	**Particle size (nm) **	**Surface area (m** ^2^ **/g)**	**Manufacturer **
**Titanium (IV) oxide **	Nano powder	21	35-65	Sigma-Aldrich Corporation
**Aluminium oxide**	Nano powder	<50	>40	Sigma-Aldrich Corporation
**Zinc oxide**	Nano powder	<100	12-25	Sigma-Aldrich Corporation
**Silicon dioxide**	Nano powder	5-15	590-690	Sigma-Aldrich Corporation

According to a literature review, few studies have compared the cytotoxicity of different nanoparticles at the same time. In 2010, Kim *et al. *[26] conducted a comparative cytotoxicity study on TiO_2_, CeO_2_, ZnO, and Al_2_O_3_. In accordance with our results they reported a dose and time dependent toxicity effect and Al_2_O_3_ nano powder was less toxic than others and ZnO had the most toxicity. Furthermore, Qiang *et al.* [28] conducted a comparative study between cytotoxicity of TiO_2_, SiO_2_, ZnO, and Al_2_O_3_ nanoparticles on human fetal lung fibroblasts. They also reported ZnO nanoparticles as the most toxic one. However, in their study all evaluations were performed after 48 h, so time dependent toxicity assessment is not possible.

Jaberiansari *et al.* [46], showed that an experimental nano hybrid MTA is severely toxic at neat concentration after 24, 48, and 72 h and also moderately toxic at 1/2 concentration on DPSCs. They did not mention the components of their experimental nano hybrid MTA. So, their comparison and judgment about nanoparticles and their percentages with other studies is not possible. 

However, because of the presence of many confounding covariates in the oral cavity, these results of *in vitro* investigations might not be thoroughly generalized to the clinical applications and more *in vivo *and clinical trials are needed for making an evidence based decisions.

## Conclusion

As the first step in comprehensive research for application of nanoparticles in dental materials especially MTA, we conclude that due to dose and time dependent toxicity of these nanoparticles, especial attention to safe administration of them is needed. It may be solved by using low-concentration incorporation with biocompatible dental materials. Based on the differential toxicity of these materials, further investigations are required to find better substitutions.
